# The control of soil-transmitted helminthiases in the Philippines: the story continues

**DOI:** 10.1186/s40249-021-00870-z

**Published:** 2021-06-12

**Authors:** Mary Lorraine S. Mationg, Veronica L. Tallo, Gail M. Williams, Catherine A. Gordon, Archie C. A. Clements, Donald P. McManus, Darren J. Gray

**Affiliations:** 1 Department of Global Health, Research School of Population Health, The Australian National University, Building 62 Mills Rd, Acton ACT, Canberra, 2601 Australia; 2 Department of Epidemiology and Biostatistics, Research Institute for Tropical Medicine, Manila, Philippines; 3 School of Public Health, University of Queensland, Brisbane, Australia; 4 Molecular Parasitology Laboratory, Infectious Diseases Division, QIMR Berghofer Medical Research Institute, Brisbane, Australia; 5 Faculty of Health Sciences, Curtin University, Perth, Australia

**Keywords:** Soil-transmitted helminths, Epidemiology, Control, The Philippines

## Abstract

**Background:**

Soil-transmitted helminth (STH) infections have long been an important public health concern in the Philippines. In this review, we describe the current status of STH infections there and highlight the control efforts undertaken to reduce STH burden.

**Main text:**

A nationwide STH mass drug administration (MDA) programme was started in 2006 but the overall STH prevalence remains stubbornly high across the Philippines, ranging from 24.9% to 97.4%. The continued increase in the prevalence may have been due to the challenges related to MDA implementation which include the lack of people’s awareness about the importance of regular treatment, misconceptions about the MDA strategy, lack of confidence on the drugs used, fear of adverse events and general distrust of government programs. There are existing water, sanitation and hygiene (WASH) programmes implemented in communities [e.g., Community-Led Total Sanitation (CLTS) program and providing toilet bowls and provision of subsidy for latrine construction] and schools [e.g., WASH in School (WINS) program], but sustained implementation is required to achieve expected outcomes. Although WASH in general is being taught in schools, integration of STH as a disease and community problem in the current public elementary school curriculum is still inadequate. The Integrated Helminth Control Program (IHCP) currently implemented in the country, which is focused on improved sanitation and personal hygiene, health education and preventive chemotherapy, will require continuous appraisal. The sustainability of this programme still continues to be a challenge.

**Conclusions:**

Despite the major efforts to control STH infections for almost two decades in the Philippines, persistently high STH prevalence has been reported across the country, which is likely due to suboptimal MDA coverage and limitations in WASH and health education programs. Sustainable delivery of integrated control approaches will continue to play a pivotal role in the control and elimination of STH in the Philippines.

**Graphic abstract:**

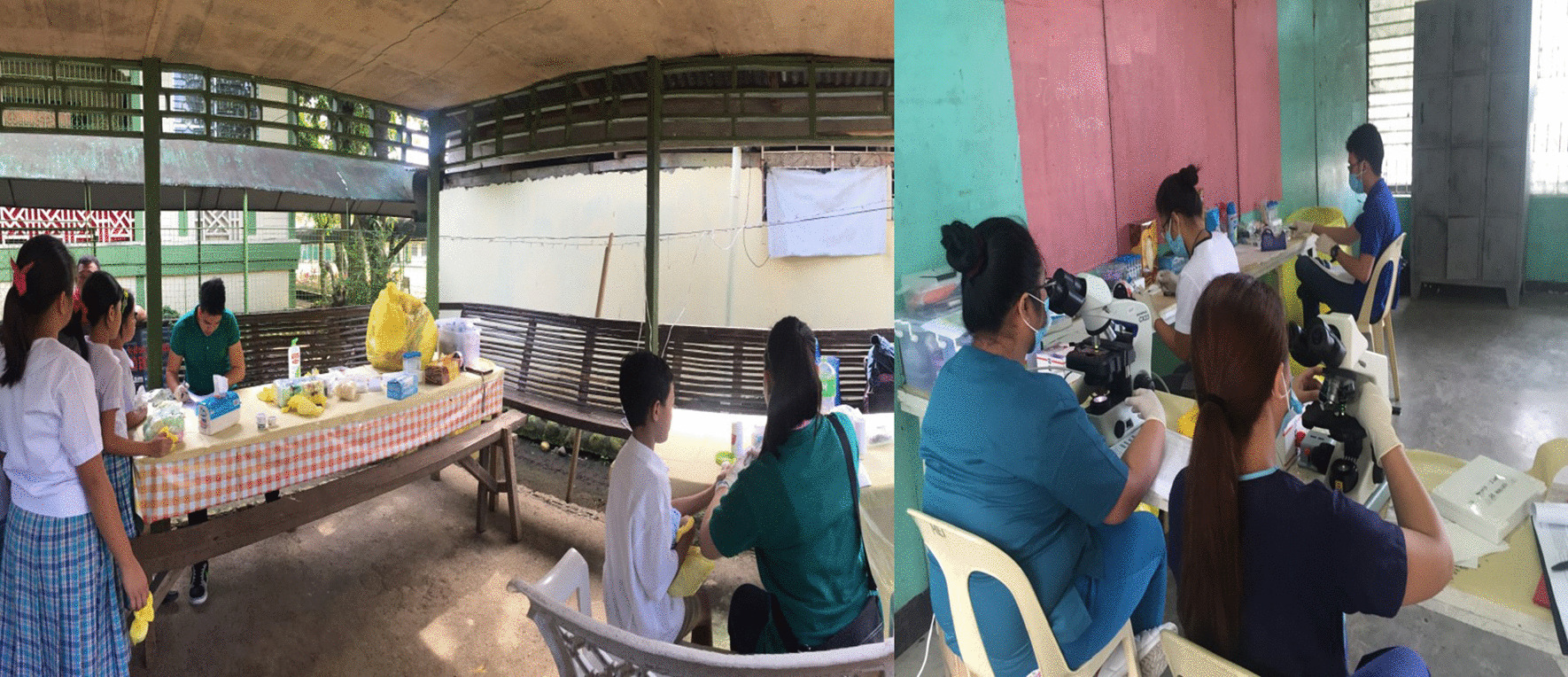

## Background

Soil-transmitted helminth (STH) infections continue to be a serious public health problem worldwide with more than 1.5 billion people estimated to be infected [1]. STH affect underprivileged communities, characterised by poor access to adequate water, sanitation and hygiene (WASH) [2, 3]; and are highly endemic in low-income countries, with the majority of infections occurring in Asia, Africa, and parts of Latin America [4]. Pre-school-aged children (PSAC) aged 2–4 years and school-aged children (SAC) aged 5–12 years are the most vulnerable to infection and have the highest prevalence and infection intensity. Available data indicate that more than 267.5 million PSAC and more than 568.7 million SAC reside in areas with intense STH transmission, requiring preventive chemotherapy [5]. The global STH burden has been estimated to range from 1.97–3.3 million disability-adjusted life years (DALYs) [6, 7].

STH infections may result in nutritional deficiency and impaired physical and cognitive development especially in children [8]. High-intensity STH infections exacerbate morbidity [9–11]. Polyparasitism (infection with multiple parasite species) has also been shown to be associated with higher mortality rates and increased susceptibility to other infections [10, 11]. The adverse impact of these infections not only affects health but also economic productivity [8, 12].

The Philippines is a lower middle-income country. In 2015, approximately 21.6% of the Philippines’ 100.98 million population were living below the national poverty line [13]. It also has some of the highest prevalence levels of STH in Southeast Asia [14]. The 2019 figures from the WHO Preventive Chemotherapy Databank indicate that about 45 million children are at risk of infection necessitating drug treatment [15].

Although several large initiatives have been launched to control or interrupt transmission, STH remains highly endemic in the Philippines [16]. In this article, we provide an overview of the current status of STH infections in the Philippines; highlight the past and current control efforts being undertaken, document the challenges and travails of the program implementation, assess their impact to reduce the STH burden and provide possible prospects for the control of intestinal worms. The availability of this information may provide the basis for planning and implementing a sustainable STH control program in the country.

This review focuses on the four most common STH parasites—*Ascaris lumbricoides, Trichuris trichiura, Necator americanus* and *Ancylostoma duodenale*. Although, *Ancylostoma ceylanicum* is emerging as an important zoonotic species of hookworm in Southeast Asia, limited information is currently available in the Philippines, and so it will no longer be discussed here.

### Search methodology

Although this is not a systematic review, the methods used for the literature review are as follows. We conducted a search of the relevant studies reporting STH prevalence in the Philippines using the PubMed, Scopus, ProQuest and Google Scholar online databases. The following words were used as keywords in the search: (“Helminthiases” OR Soil-transmitted helminths” OR “STH” OR “*Ascaris lumbricoides*” OR “Trichuris trichiura” OR “*Ancylostoma spp.”* OR “*Necator americanus*” or “Roundworm” OR “Whipworm” OR “Hookworm”), AND (“Epidemiology”) AND (“Philippines”). There was no restriction on the year of publication. Articles identified by the search criteria were initially screened by title and abstract content and those that did not investigate prevalence or intensity in at least one of the three STH were excluded. Full text screening included observational (cross-sectional, case control, longitudinal/cohort) studies or controlled trials which reported the baseline prevalence. Data extraction included study area, year the study was conducted, year the study was published, type of study (cross-sectional, case–control or longitudinal/cohort), sample size, study population, prevalence and intensity of each STH and the diagnostic methods used.

**Fig. 1:**
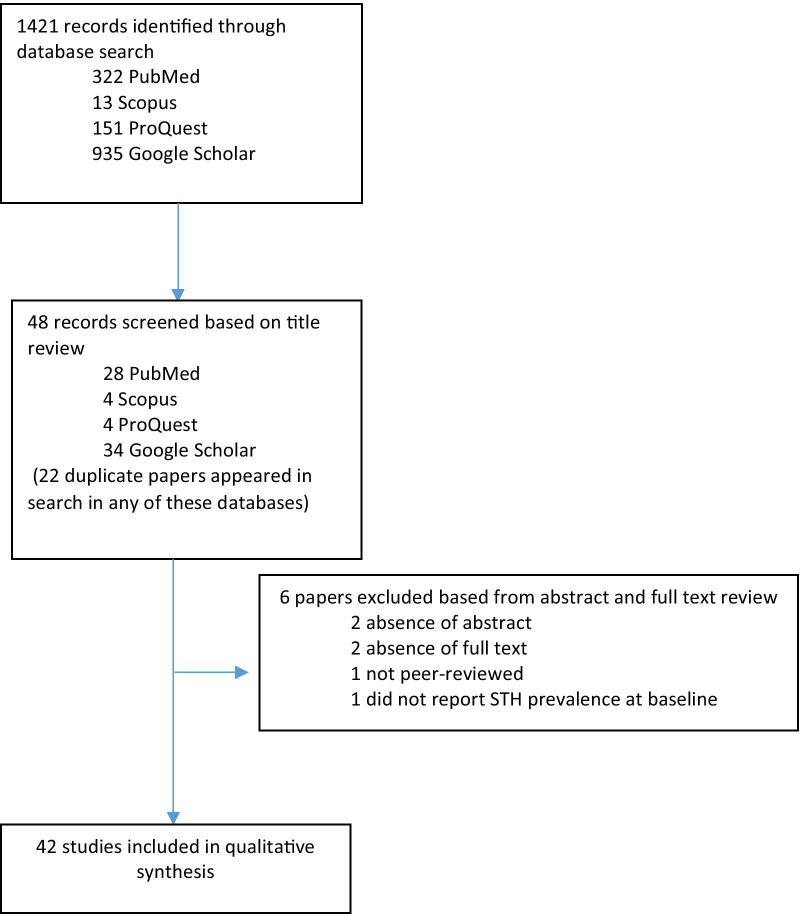
Study selection

Based from the literature search, a total of 1421 records were identified through database searching [PubMed (*n* = 322); Scopus (*n* = 13); ProQuest (*n* = 151), and Google Scholar (*n* = 935)]. A total of 48 papers were screened based on title review, of which 6 papers were then excluded, bringing the final total to 42 papers included in the qualitative synthesis (Fig. 1).

### Epidemiology of STH infections in the Philippines

Numerous studies have been conducted since the 1970s in the Philippines to determine the prevalence and intensity of STH infections. Table 1 shows a summary of studies identified. Differences in the methods of STH diagnosis were evident across these studies over time, with the formalin-ether concentration (FEC) method frequently being used in the early period (1970–1998). The Kato-Katz (KK) technique, however, was used increasingly in the later years and employed as the primary diagnostic method in the national surveys to monitor the STH control program.

**Table 1:** Key outcomes of studies investigating STH infections in the Philippines

Year of the Survey conducted	Study description	Sample size	Study population	Diagnostic method	Key infection outcomes (STH Prevalence)					Reference/Year of publication
					*Ascaris lumbricoides*	*Trichuris trichiura*	Hookworm	Overall prevalence	Heavy infection	
Before the launch of the IHCP (i.e., programme components include nationwide MDA, WASH and health education)										
1974–1975 (baseline survey)	A comparative study on the control and eradication of ascariasis in rural community of Salvacion, Palo, Leyte	606	All ages	FET	84.4%	-	-	-	-	Cabrera et al., (1975) [95]
1977	A cross-sectional survey on intestinal parasites conducted in Municipalities of Talibon and Trinidad, Northern Bohol, Philippines, with emphasis on schistosomiasis	1694	All ages	FET	44.5%	57.8%	71.4%	-	-	Carney et. al., (1980) [96]
1979 (baseline survey)	A comparative study on the effect of mass treatment of the entire community and selective treatment of children on the total prevalence STH in communities of Ordovilla and San Narciso, Mindoro Oriental	552	All ages	Not mentioned	71.6%	81.8%	20.1%	-	-	Cabrera et al., (1983) [97]
1986	A cross-sectional survey to determine the prevalence intestinal parasites in Napsan Palawan	365	All ages	FET	34.8%	25.2%	34.8%	-	-	Oberst et al., (1987) [98]
Not reported	A cross-sectional survey to examine the prevalence of schistosomiasis and common intestinal parasites among the residents of seven villages in Agusan del Norte Province, Mindanao, Philippines	1920	All ages	Direct Smear, FET and Harada-Mori stool culture	45.0%	48.0%	83.0%	-	-	Carney et.al. 1987 [99]
1980–1984	A survey on intestinal parasites in some patients seen at San Lazaro Hospital, Manila	3687	All ages	FET	30.0%	46.0%	29.0%	-	-	Cross et al., (1989) [100]
1994	A cross-sectional survey of intestinal parasitic infections in San Narciso, Victoria, Oriental Mindoro	242	All ages	FET	43.8%	48.8%	11.6%	-	-	Chigusa et al., (1997) [101]
1998	A small-scale survey of intestinal parasite infections among children and adolescents in Legaspi city	64	Ages 3–20 years	FET	40%	51.0%	23.0%	-	-	Lee et al., (2000) [102]
Not reported	This study was conducted in a rural agricultural area in Siniloan, Laguna, Philippines to investigate the relationship between helminthiasis infection and nutritional status	142	SAC	KK	40.30%	71.40%	-	-	-	Yamamoto et.al, 2000 [103]
1998 (baseline survey)	A study on the comparison of the efficacy of single doses of albendazole, ivermectin, and diethylcarbamazine alone or in combinations against *A. lumbricoides* and *T. trichiura spp.* among SAC in public elementary schools in Biñan, Laguna Province	784	SAC	KK	67.40%	96.8%	0.38%	64.2%	67.4%	Belizario et al., 2003 [18]
2000	A baseline assessment of intestinal parasitism on schoolchildren (Kinder and Grade 1 pupils) in selected public elementary schools in 6 provinces across the Philippines (Luzon, Visayas and Mindanao)	1871	PSAC and SAC	KK	46.30%	52.27%	4.6%	66.8%	9.9%	Belizario et al., 2005 [19]
2001 (baseline data)	Cross-sectional survey to determine the prevalence and intensity of STH infection among schoolchildren in Monkayo, Compostela Valley	173	SAC	KK	4.0%	16.8%	35.3%	48.6%		Belizario, et al., 2004 [23]
2002	A cross-sectional study in rural rice community in Leyte. Blood samples were collected for anemia determination and cognitive testing was performed. Stool samples were also collected and tested for *Schistosoma japonicum* and geo-helminth infection	322	Ages 7–18 years	KK	76.2%	94.6%	76.2%	-	-	Olson et al., (2009) [104]
2002	A small-scale survey to investigate the status of intestinal protozoa and helminth infections in Roxas City, Mindoro, Philippines	301	All ages	FET	51.2%	27.6%	8.0%	-	-	Kim et al., (2003) [105]
2002	A survey conducted to determine the infection status of intestinal parasites in children living in residential institutions in Metro Manila, the Philippines	172	Children	FET	36%	44.8%	7.0%	-	-	Baldo et al., (2004) [106]
2004	National survey conducted in 2004 among PSAC in 17 regions of the country	6358	PSAC	KK	48.7%	42.2%	1.7%	66.4%	-	de Leon et al., 2005 (cited at [20])
2005	Prevalence survey of intestinal parasites among schoolchildren in a coastal rural area of Maragondon, Cavite, Southern Luzon, Philippines	259	SAC	KK	66.4%	14.7%	21.2%	-	-	Cauyan et al. (2008) [107]
2005–2007^a^	National baseline prevalence surveys of schistosomiasis in Visayas (11 provinces) and Mindanao (22 provinces) regions carried out between 2005–2007	27 771	All ages	KK	20.7–34.6%	16.3–45.9%	7.9–11.4%	-	-	Leonardo et al., 2008 (25)
2005–2007^a^	This study used the STH data collected from the national schistosomiasis survey in the Philippines from 2005–2007. This study aimed to quantify the association between the physical environment and the prevalence of *A*. *lumbricoides*, *T*. *trichiura* and hookworm infections in the Philippines	35 573	All Ages	KK	23.7% (Luzon)	27.9% (Luzon)	4.5% (Luzon)	-	-	Magalhães et al., 2015 [16]
					38.4% (Visayas)	53.6% (Visayas)	18.0% (Visayas)	-	-	
					21.2% (Mindanao)	16.8% (Mindanao)	11.3% (Mindanao)	-	-	
Not reported	Cross-sectional study conducted among children aged 7–18 years in Leyte, Philippines to examine the independent effect of infection with each of four helminths (*Ascaris lumbricoides, Schistosoma japonicum, Necator americanus, and Trichuris trichiura*) on cognitive function	319	SAC	KK	73.9%	92.2%	45.8% *(N. americanus)*	-	-	Ezeamama, et al. 2006 [108]
2006	A baseline parasitological survey conducted in 6 sentinel provinces (Bulacan and Camarines Sur in Luzon; Negros Occidental and Leyte in Visayas, and Compostela Valley and Surigao del Norte in Mindanao) in the country	3373	SAC	KK	38.6%	38.5%	3.8%	54.0%	23.1%	Belizario et al., 2009 [21]
2006	Monitoring school-based control of intestinal helminthiasis in selected school districts in Cavite Province (Calabarzon Region)	700	SAC	KK	42.6%	49.1%	0.6%	61.4%	36.4%	Belizario et al. (2013) [22]
2008^a^		732			27.4%	39.8%	-	47.7%	24.6%	
After the launch of the IHCP (i.e., programme components include nationwide MDA, WASH and health education)										
2007	A cross-sectional parasitological survey conducted to determine the impact of MDA on STH on the morbidity of SAC in selected provinces in the western Visayas. The surveys were carried out at three different time points: in 2007, 2008 and 2009	1230	SAC	KK	40.7%	63.8%	2%	71.1%	40.5%	Belizario et al., 2014 [28]
2008		1349			25.4%	45.7%	1.9%	52.3%	22.5%	
2009		1211			17.2%	38.8%	3.6%	44.3%	14.5%	
2007	A cross-sectional parasitological survey conducted in 2007, 2009, and 2011 to monitor the impact of a mebendazole MDA initiative for STH control among SAC in the Western Visayas Region of the Philippines	1230	SAC	KK	40%	65.0%	-	70.0%	-	Sanza et al., 2013 [29]
2009		1243			18%	40.0%	-	44.1%	-	
2011		1037			25%	40.0%	-	45.5%	-	
2007–2008	The last two phases of the national baseline prevalence survey of schistosomiasis conducted between 2007 and 2008 in Luzon and Maguindanao Province	3541	All Ages	KK	15.3–18.4%	18.1–20.8%	0.3–4.6%	-	-	Leonardo et al., 2012 [24]
2008	Survey of stool samples obtained from the residents of Municipality of Katipunan, Zamboanga Del Norte to determine the status of intestinal capillariasis	205	All ages	FET	22.3%	31.1%	16.5%	44.2%	-	Belizario et al. (2010) [40]
2009	Parasitology survey conducted to determine STH infections and other intestinal parasitic infections among SAC in indigenous people communities in Davao del Norte	572	SAC	KK	20.1%	11.7%	11.9%	34.1%	5.9%	Belizario et al., (2011) [30]
2009	A follow-up survey to monitor the impact of an integrated helminth control program conducted in 6 sentinel provinces (Bulacan and Camarines Sur in Luzon; Negros Occidental and Leyte in the Visayas, and Compostela Valley and Surigao del Norte in Mindanao)	2,474	PSAC		30.9%	31.4%	1.1%	43.7%	22.4%	Belizario et al., 2015 [26]
		2,751	SAC	KK	27.7%	33.3%	1.9%	44.7%	19.7%	
2011	Prevalence survey to determine STH infections and its association with haemoglobin levels among Aeta schoolchildren of Katutubo Village in Planas, Porac, Pampanga	195	SAC	KK	84.1	94.4%	21.5	97.4%	82.6% (M-H)	Ng et al., 2014 [31]
2011	A diagnostic study of intestinal helminths in Northern Samar Province. Faecal samples were collected from individuals and examined by real-time PCR (qPCR) assay	545	All ages	qPCR	58.17%	-	48.07% (*Ancylostoma* spp.)	-	-	Gordon et al., 2015 [45]
2011–2012	A cross-sectional survey conducted to determine prevalence and intensity of infection in selected secondary schools in two provinces (Cavite and Guimaras)	633	14–15 years old	KK	19.7%	21.5%	0.2%	31.3%	7.7% (M–H)	Belizario et al., (2014) [41]
2012	A cross-sectional survey conducted in 18 rural barangays in Northern Samar to determine the prevalence of single and multiple species helminth infections and the underlying risk factors of acquiring one or more parasite species	6976	All ages	KK	36.5%,	61.8%,	28.4%,	75.8%	-	Ross et al., 2017 [42]
2013	A cross-sectional survey conducted to determine the prevalence and intensity of STH infections and determine the nutritional status of PSAC and SAC in two villages in Southern Leyte that benefitted from CLTS	316	PSAC and SAC	KK	15.8%	19.9%	1.2%	27.9%	7.9% (M–H)	Belizario et al. (2015) [32]
2013	A cross-sectional survey conducted to determine the status of STH in SAC in Masbate after a decade of implementation of an integrated helminth control programme	1224	PSAC	KK	59.0%	54.0%	2%	72.0%	41%	Belizario et al., 2016 [33]
2013	A cross-sectional survey carried out in five rural villages in Northern Samar, the Philippines. Data on dietary intake, nutritional status, and intestinal parasites were collected	693	SAC	KK	54.40%	71.4%	25.30%	79.6%	-	Ross et al., 2017[34]
2013	A cross-sectional survey among SAC in 5 schistosiamis-endemic villages in Northern Samar, the Philippines to determine the interactions between childhood malnutrition and parasitic helminth	693	SAC	KK	54.4%	71.4%	25.3%	-	-	Papier, et al., 2014 [35]
2013–2015	A cross-sectional survey conducted to determine the prevalence of STH infections in selected barangays/villages in the provinces of Cavite, Guimaras, Iloilo, Negros Occidental, and Davao del Norte	1732	PSAC	KK	19.1%	12.1%	-	24.9%	10.3% (M–H)	delos Trinos et al., 2019 [37]
2014	A cross-sectional survey of STH infection in Laguna Province carried out in 2014. Faecal samples were collected from 263 SAC and examined using the KK method and real time PCR (qPCR)	263	SAC	KK	20.50%	23.6%	-	33.8%	-	Mationg et al., 2017 [36]
				qPCR	60.8%	38.8%	6.8% (*Ancylostoma* spp. (4.6%) and *N. americanus* (2.2%)	78.3%	-	
2015	A cross-sectional survey to determine the prevalence of STH and schistosomiasis in a sample of families in four villages in Leyte, Philippines	338	PSAC/SAC	KK	35.1%	55.1%	2.7%	65.0%	8.6%	Liwanag et al., 2017 [38]
		261	Adults		27.0%	50.5%	10.0%	64.0%	4.0%	
2017	Prevalence of Soil Transmitted Helminths and Associate Transmission Factors among School Children in a Selected Barangay in Trece Martires City, Cavite	108	PSAC/SAC	KK	29.6%	7.4%	18.5%	37.0%	-	Soriano, et al., 2019 [39]
2017	A cross-sectional survey to determine the prevalence of intestinal parasites including the associated risk factors among food vendors and slaughterhouse worker in Metro Manila	91	Adults	FET	54.9%	30.4%	4.4%	-	-	Lirio, et al., 2017[43]
2018	Cross-sectional survey to determine the prevalence and intensity of STH infection among the indigenous communities in three barangays in Tigaon, Camarines Sur, Philippines	317	All ages	Sucrose centrifugal floatation method	41.9%	5.4%	-	44.5%	-	Delaluna, et al.,2020 [44]

KK, Kato-Katz; qPCR, Real-time Polymerase Chain Reaction; FET, Formalin-ether technique; M–H, Moderate to Heavy Intensity infections; CLTS, Community-Led Total Sanitation; PSAC, Pre-school children; SAC, School-aged children; IHCP, Integrated Helminth Control Program

^**a**^Results of people included in the survey undertaken in 2007 are impacted by the implementation of IHC

As indicated by studies conducted from the 1970s–2018, STH infections have been and still are important public health problems in the Philippines. The epidemiological patterns of STH infections and their prevalence are comparable to those reported in other endemic countries around the world, with the highest infection prevalence recorded in PSAC and SAC [17]. These age groups are at greater risk as these children are frequently exposed to STHs in outdoor settings.

Historically, the prevalence of any STH infections and heavy intensity infections in children aged 1–12 years before the implementation of the Integrated Helminth Control Program (IHCP) of the Department of Health ranged from 48.6–66.8% to 9.9–67.4%, respectively [18–23] (Table 1).

STH data from the national schistosomiasis survey undertaken from 2005 to 2008 across all ages showed wide-scale distributions of STH infection across three principal geographical divisions of the country, with *A. lumbricoides and T. trichiura* particularly prevalent in the Visayas [16, 24, 25].

In 2009, a follow-up assessment of the nationwide STH prevalence surveys for PSAC in 2004 [20] and for SAC in 2006 [21] was conducted to assess the impact of the IHCP [26]. The prevalence of any STH for PSAC was 43.7% (vs 66% in the 2004 survey) and 44.7% (vs 54% in the 2006 survey) for SAC [26]. These were significantly lower than reported in the earlier two surveys. The prevalence of heavy-intensity STH infections for PSAC in 2009 was 22.4% (comparison with the 2004 survey was not possible as the overall prevalence of heavy intensity infection was not reported), and 19.7% (vs 23.1% in 2006 survey) for SAC with a 14% reduction [26]. Although, reductions in infection prevalence were evident, the estimated prevalence of STH in the PSAC and SAC populations still did not meet the 2020 targets of less than 20% cumulative prevalence and less than 1% heavy intensity STH infection as defined by WHO to demonstrate morbidity control [27, 48].

Other studies employing parasitological surveys conducted at several time points (2006–2011) to monitor the impact of the school-based MDA among SAC also showed similar trends [22, 28, 29]. Results from these surveys demonstrated reductions in STH prevalence after several rounds of MDA; however, the reported overall prevalence for any STH (ranging from 44.3 to 47.7%) and heavy intensity infections (ranging from 14.5 to 24.6%) were still high at the follow-up surveys [22, 28, 29], again indicating that the prevalence had not been reduced to the target levels for morbidity control defined by WHO (Table 1).

Data from other studies identified after the launch of IHCP across the Philippines in 2007–2018 among PSAC and SAC showed persistently high levels of STH prevalence (Table 1) [30–39]. The prevalence of any STH reported from these studies ranged from 24.9 to 97.4% (by KK) and the prevalence of moderate to heavy intensity infections ranged from 5.9 to 82.6%. *A. lumbricoides* and *T. trichiura* remained the most prevalent STH with prevalence ranging from 15.8–84.1% to 7.4–94.4%, respectively, while the hookworm prevalence tended to be lower ranging from 1.2 to 25.3% [30–39] (Table 1). One study, however, in 2011, using the molecular diagnostic quantitative real time polymerase chain reaction (qPCR) showed a hookworm (*Ancylostoma spp.*) prevalence of 48.1% [45]. Co-infections of individuals with *A. lumbricoides* and *T. trichiura* were also commonly observed in several studies [26, 31, 33, 36, 45].

The KK method, recommended by the WHO due to its ease of use in the field and low cost [46], is primarily employed for assessing the government treatment program for STH control. However, differences in STH prevalence have been reported between KK and other diagnostic methods. In a study conducted in 2014 in the province of Laguna, a large discrepancy in the prevalence between KK and qPCR was noted in the detection of any STH infection (33.8% by KK vs 78.3% by qPCR), *A. lumbricoides* (20.5% by KK vs 60.8% by qPCR) and *T. trichiura* (23.6% by KK vs 38.8% by qPCR). There were also hookworm infections [6.8% prevalence; comprising *Ancylostoma* spp. (4.6%) and *Necator americanus* (2.2%)] detected using qPCR that were judged as negative by KK [36]. The true prevalence of hookworm infections can be considerably underestimated because the rapid lysis of hookworm eggs necessitates a rapid turn-around in KK slide preparation and reading [36, 45, 47], a process often difficult to achieve under field conditions. In addition, the eggs of hookworm species are indistinguishable morphologically, which presents a further challenge in terms of correct identification [45].

### STH control in the Philippines

The main strategy for STH control advocated by WHO focuses on large-scale preventive chemotherapy using albendazole or mebendazole for at-risk populations with the target of treating at least 75% of PSAC and SAC by 2020 [48]. Until the recent launch of the Neglected Tropical Diseases (NTDs) 2030 roadmap, the WHO recommends that PSAC, SAC, and women of reproductive age (aged 15–49 years, including pregnant women in their second and third trimester) receive regular treatment [49]. Additionally, the guidelines include young children (aged 12–23 months) and adolescent girls (aged 10–19 years) [49] but exclude the previous recommendation of treating adults in high-risk occupations [50]. The WHO recommends annual MDA of young children, PSAC, SAC, adolescent girls and women of reproductive age in areas where the prevalence of STH is between 20 and 50% and semi-annual if the prevalence is above 50%. For pregnant women, the treatment interval was not defined [49]. In addition to preventive chemotherapy, the WHO has highlighted water, sanitation and hygiene (WASH) as important components of STH control [48, 49].

The IHCP was launched in 2006 to provide policy directions for the control of STH and other helminth infections [20, 51]. The program, which follows the strategies endorsed by WHO for STH control, has chemotherapy with albendazole or mebendazole as the primary strategy for STH control and targets children aged 1–12 years and other high-risk group such as pregnant women, adolescent females, farmers, food handlers, and the indigenous population. The control program is also supplemented with the installation of water and sanitation facilities and health promotion and education approaches [20, 46].

### Preventive chemotherapy

The semi-annual MDA for PSAC is primarily conducted in the community setting by local barangay (village) health units, trained barangay health workers, and day care workers as part of the Garantisadong Pambata or “Healthy Children” (a program that delivers a package of health services for PSAC) while the MDA for SAC is overseen and implemented by the Department of Education (DepEd) [20]. MDA in public elementary schools is administered by teachers under the direction of health workers every first and third quarter of the school year [20]. In 2016, the DOH issued new guidelines to include deworming in secondary schools (children up to 18 years of age) [52].

The first nationwide semi-annual MDA was conducted among children aged 1–12 years in 2006 [20], reporting a deworming coverage of 82.8% of the 6.9 million PSAC and 31.5% of the 6.3 million SAC [53]. However, MDA deworming coverage from 2009–2014 went down considerably (range 59.5–73.9%), a figure consistently below the 75% benchmark recommended by the WHO [54]. The low deworming coverage may have been due to a lack of awareness of the importance of regular treatment [55], misconceptions about the MDA strategy [56, 57], lack of confidence in the drugs used [58], and fear of adverse events [55, 56, 58–60]. There have been reports of fear of birth defects as a reason for refusing STH treatment in pregnant women [61]. Moreover, the supply of MDA drugs and logistical issues have been identified as major shortcomings encountered during the nationwide MDA implementation [54].

In 2015, the DOH, in partnership with DepEd, conducted the first National School Deworming Day (NSDD) that aimed to deworm approximately 16 million SAC (grades 1 to 6) enrolled in all public elementary schools in one day [62]. This school-based initiative resulted in a national deworming coverage of 81%, which was higher than the previous years [54]. However, false information circulating in the community on deaths among children following deworming and the use of expired drugs caused mass hysteria and panic, resulting in an increase of reports of adverse events following MDA (AEFMDA) in Zamboanga Peninsula on the island of Mindanao [63]. However, a case-control study, showed that being an AEFMDA case was associated with no history of previous deworming [63].

In 2017, a new dengue vaccine was introduced by the DOH and given to some 800 000 school children. The provision of this vaccine raised major safety issues and resulted increased distrust in the DOH programs, including the MDA program [64, 65]. As a result, the deworming coverage decreased from 81 and 73% among PSAC and SAC in 2017 to 63% and 52%, respectively in 2018, and to 60% and 59%, respectively, in 2019 [15].

Additionally, in view of the current global COVID-19 (coronavirus disease 2019) pandemic, the DOH issued the Department Memorandum Number 2020–0260 or the “Interim Guidelines on Integrated Helminth Control Program and Schistosomiasis Control and Elimination Program during the COVID-19 pandemic” on June 23, 2020, prescribing the suspension of MDA until further notice. Due to school closures, routine deworming of children aged 1–18 was administered in the community either through house-to-house visits or fixed site distribution of drugs while maintaining physical distancing and appropriate infection prevention control measures against COVID-19 [66]. The restrictions on the movement of people and the public anxiety due to COVID-19 pandemic may however, lead to lower treatment coverage.

#### WASH

WASH is one of the key interventions outlined by the IHCP for STH control [20, 46]. It is a program that involves multiple government agencies including the DOH, Department of Interior and Local Government (DILG), local government units (LGUs) and DepEd. Among the WASH programs in the community are provision of access to safe water which is spearheaded by the LGUs with support of DILG [67] and sanitation improvements implemented by the DOH with the help of the LGUs in providing toilet bowls and subsidies for latrine constructions [68, 69]. Meanwhile, WASH programs in public elementary schools is supervised by the DepEd, in partnership with the DOH.

Recent data from the 2017 National Demographic Health Survey by the Philippines Statistics Authority (PSA) shows that 95% of Filipino households obtain their drinking water from an improved source, the greatest proportion (43%) sourcing their water from bottled water, with only 26% obtaining it from a piped sourced [70]. A quarter of Filipino households still use unsatisfactory sanitation [70]; and about 4.5% of the population openly defecate, a practice twice as prevalent in rural areas (6%) than in urban areas (3%) [70].

Other reports have shown that the provision of sanitation facilities alone did not guarantee their utilization nor did they result in improving sanitation and hygiene practices [32, 68, 69]. The most frequently cited reason for not improving sanitation among households that lack a toilet include technical barriers (i.e., lack of space in the home for toilets or around the home for septic tanks, and other geographical considerations such as soil conditions and proximity to waterways), land ownership and lack of funds [71, 72].

The DOH through the Development of Sustainable Sanitation in East Asia – Philippines Programme, adopted the Community-Led Total Sanitation (CLTS) approach in 2007 [68, 73]. CLTS is under the umbrella concept of total sanitation, which includes a range of behaviours such as stopping open defecation practices, ensuring that everyone uses a sanitary toilet, frequent and proper hand washing, hygienic handling of food and water, safe disposal of animal and domestic waste, and creation and maintenance of a clean and safe environment [68, 69]. To ensure sustainability of the CLTS approach, the ODF status of the villages should be continuously monitored even after the termination of the CLTS activities. Nevertheless, some studies have shown high STH prevalence in communities that have attained ODF status after the CLTS was implemented [32, 33]. This may have been due to the non-utilization of sanitary facilities, the possible reversion to open-defecation, and low MDA coverage [32].

The WASH programs implemented in schools follow the policies issued by the DOH and the DepEd. In 1998, the DOH issued the Implementing Rules and Regulations (IRR) of School Sanitation and Health Services of the Code on Sanitation of the Philippines (PD No. 856) [74]. This IRR specifies the rules and regulations on school sanitation and satisfactory sanitary facilities, which include toilets, water supply, and the care and maintenance of these facilities [74]. However, assessments of the DepEd’s implementation of this program in selected provinces showed that the guidelines have not been strictly implemented and there was insufficient budgetary support [57, 75–77]. Thus, monitoring and evaluation will remain critical to ensure the sustainability of the DepEd’s implementation of the WASH program.

In addition, to institutionalize good health practices among students, the DepEd issued Department Order (D.O.) No. 56, s. 2009 entitled “Immediate construction of Water and Hand Washing Facilities in All Schools for the Prevention of Influenza A (H1N1)” and D.O. No. 65, s. 2009 entitled “Implementation of the Essential Health Care Program (EHCP) for the school children” [78, 79]. Although the first program was aimed at preventing the spread of H1N1, this was also relevant for STH control. The latter follows the Fit for School approach and focuses on three evidence-based school health interventions: handwashing with soap, tooth brushing with fluoride toothpaste as a daily group activity, and semi-annual MDA for STH [78, 80]. In 2016, the EHCP was now integrated into the WASH In Schools (WINS) program. It was expanded to include provision of water, sanitation, food handling and preparation, hygiene improvement (e.g., menstrual hygiene management), deworming and health education [79].

### Heath education

While in general WASH, is included in the elementary school curriculum [79], the inclusion of STH infections as a disease and public health problem is still lacking. A recent study conducted in selected public elementary schools in the province of Cagayan reported that health education related to WASH is available to all students regardless of their grade level and type of school and it is also integrated in several subjects and is pervasively promoted (i.e., materials that promote health education are visually present in classrooms, WASH areas and the entire school) [57]. However, the same study recommended the need for teachers to receive training on STH and deworming to develop a deeper understanding of the parasites and a greater appreciation of STH as a public health problem including: topics related to STH transmission, risk of infection, risk of open defecation and mode of reinfection after deworming being introduced into the school curriculum [57].

The relationship between health education and treatment acceptance has also been demonstrated in other studies [56, 60] which indicate that intensified health education and promotion (to improve STH knowledge and to correct MDA misconceptions on treatment and benefits) could increase MDA treatment participation and acceptance [56, 60].

Additionally, the importance of health education to influence good sanitation related behaviours has been identified as one of the crucial components of WASH implementation [33, 60]. As indicated in previous studies, open defecation does not necessarily arise from the lack of latrine access [32, 33]. Factors such as the habit of open defecation and non-use of sanitary facilities may influence open defecation outcomes [68, 69]. In another study, poor sanitation facilities have been linked to a high risk of functional illiteracy among SAC in the Visayas region [81]. Thus, the importance of incorporating health education and promotion strategies aimed at improving defecation and hygiene practices, and acceptance and appropriate use of these sanitary infrastructure, are needed to sustain the uptake of WASH interventions.

### Future directions

Data collected over the past two decades indicate that the prevalence and intensity of STH infections remain high among children aged ≤ 12 years in the Philippines, despite the various efforts of the Philippine government. Identification of barriers and challenges in MDA participation and treatment compliance is required to ensure high MDA coverage. It is also noteworthy to consider the efficacy of the two drugs (albendazole and mebendazole) currently used by the STH control program, as alarmingly high *T. trichiura* infections have been reported in some recent studies in the Philippines [33, 34, 42]. Both drugs are reported to have low efficacy against *T. trichiura,* with pooled cure rates of 30.7% and 42.1%, and egg reduction rates of 49.9% and 66.0%, for albendazole and mebendazole, respectively [82]. This may have critical implications in areas where *T. trichiura* is prevalent, given the minimal treatment impact of both drugs. Chemotherapy is effective at reducing the level of infection and reducing worm burdens of infected individuals below the morbidity threshold but as well as variable efficacy across the STH species. It is of note that the available drugs do not prevent reinfection, which can happen immediately after treatment. Thus, new drugs and drug combination strategies may be needed in the future [83].

Currently, there is no mandated MDA treatment for adults in the Philippines. The IHCP focuses only on children aged 1–18 years, and selective deworming of other at-risk groups such as pregnant women, adolescent females, farmers, food handlers, and the indigenous population [46]. However, recent mathematical modelling [84–86] and a systematic review and meta-analysis [87] show that expanding the deworming programmes community-wide, to encompass all age groups, is likely to reduce the prevalence of STH in the high-risk schoolchildren group. Scaling up from targeted drug administration to community-wide MDA may, however, have important economic implications for the STH control program since an increase in resources would be needed. Nevertheless, the effective mass treatment campaign for lymphatic filariasis conducted in the Philippines highlights the feasibility of delivering community-wide treatment [52].

As a result of the ongoing COVID-19 pandemic, the school-based MDA campaign for STH across the Philippines has been halted and so resurgence in STH infections is expected. Recent mathematical modelling showed that delays in MDA in high STH prevalence settings may mean that the 2030 target for STH elimination as a public health problem (EPHP) (defined as reaching < 2% prevalence of moderate-to-high intensity infections in SAC [88]) may not be achievable, although mitigation strategies (i.e. higher MDA coverage,  > 75%) to make up for the missed MDA rounds will be beneficial [89]. Therefore, more sustainable control strategies to augment MDA are urgently needed to combat STH infections in the Philippines.

In addition to MDA, transmission interruption also requires changes in hygiene behaviour, access to safe water and improvement of sanitation facilities through effective WASH and CLTS programmes. Somewhat discouragingly, however, there have been reports of sanitary facilities provided by LGUs not being fully utilized in some communities, reflecting existing challenges in WASH implementation [68, 69, 71, 72]. Furthermore, high STH prevalence has been reported in communities who have attained ODF status after CLTS implementation due to reversion to open defecation behaviour and low MDA coverage [32]. Cultivating knowledge and awareness of STH and improving hygiene behaviour is an important approach in lowering the individual risk of infection, essentially providing a low-cost supplement to MDA and WASH programmes.

Health education delivered in schools may help strengthen and improve both student and parental knowledge and awareness about STH in general, including the perceived benefits of deworming. An example of a recent and highly successful health education intervention delivered in schools is the “Magic Glasses” program. This is a short cartoon-based intervention designed to educate students on STH infection and prevention provided proof of principle that health education can improve knowledge and influence behaviour related to STH infection [90]. This program was first used on Chinese primary school children in Hunan Province which reduced the incidence of STH infection by 50% (odds ratio = 0.5, 95% confidence interval: 0.35–0.7, *P* < 0.0001) in intervention schools compared with control schools [90]. This has been adapted and rigorously tested in the Philippines [91] and in Vietnam; and is currently being developed for the Lower Mekong region including its adaptation to combat carcinogenic *Opisthorchis* liver fluke infections. Experiences in several Asian countries particularly Japan, Republic of Korea and Taiwan Province of China have shown that elimination of STH infections is possible with MDA, appropriate sanitation and hygiene education as part of national control programmes through a school-based approach and triangular cooperation among government agencies, non-government organizations and scientific experts [92–94].

There are several programmes incorporating STH control operating in the Philippines such as the WASH/EHCP or WINS implemented in schools and the CLTS implemented in communities. However, for greater chances of sustainability, there is a need for improved coordination among organizations implementing the program. Thus, a decentralised program and a multi-party endeavour such as that undertaken for STH control in the Philippines can only succeed with long term collaboration, cooperation and buy-in from the local government. There is a need for governmental support in the procurement and distribution of drugs and in prioritizing other components of the control program such as activities that improve environmental sanitation and health education to accelerate progress towards the 2030 EPHP goal [88]. With the challenges of the COVID-19 pandemic, these activities need to be continued and be integrated with the ongoing COVID-19 prevention efforts. Otherwise, compromising the already challenged STH control programme may result in severe long term public health outcomes.

## Conclusions

The Philippines has undertaken major efforts to control STH infections for almost two decades. Nevertheless, persistently high STH prevalence has been reported across the country, which is likely due to suboptimal MDA coverage and limitations in WASH and health education programs. The National government should now consider strengthening school-based MDA and expansion to community-wide MDA; close monitoring of drug effectiveness during MDA campaigns and investigating the development and use of new antihelminthic drugs or drug combinations; and the sustainable delivery of WASH and health education as an integrated method of attack for future STH control in the Philippines.

## Data Availability

Not applicable.

## Abbreviations

AEDMDA: Adverse events following MDA
CLTS: Community-led total sanitation
COVID-19: Coronavirus disease 2019
DALY: Disability-adjusted life year
DepED: Department of Education
DILG: Department of Interior and Local Government
DOH: Department of Health
EPHP: Elimination as a public health problem
EHCP: Essential health care program
FET: Formalin-ether technique
IHCP: Integrated helminth control program
KK: Kato-Katz
MDA: Mass drug administration
NTDs: Neglected tropical diseases
PSAC: Pre-school children
qPCR: Real-time polymerase chain reaction
SAC: School-aged children
STH: Soil-transmitted helminths
WASH: Water, sanitation and hygiene
